# Thyroid Peroxidase Activity is Inhibited by Phenolic Compounds—Impact of Interaction

**DOI:** 10.3390/molecules24152766

**Published:** 2019-07-30

**Authors:** Ewa Habza-Kowalska, Agnieszka A. Kaczor, Justyna Żuk, Dariusz Matosiuk, Urszula Gawlik-Dziki

**Affiliations:** 1 Department of Biochemistry and Food Chemistry, University of Life Sciences, Skromna Str. 8, 20-704 Lublin, Poland; 2 Department of Synthesis and Chemical Technology of Pharmaceutical Substances, Faculty of Pharmacy with Division of Medical Analytics, Medical University of Lublin, 4A Chodzki St., PL-20093 Lublin, Poland; 3 School of Pharmacy, University of Eastern Finland, Yliopistonranta 1, P.O. Box 1627, FI-70211 Kuopio, Finland

**Keywords:** thyroid peroxidase (TPO), dietary polyphenols, molecular modeling, interactions

## Abstract

The aim of this study was to estimate the mode of thyroid peroxidase (TPO) inhibition by polyphenols: Chlorogenic acid, rosmarinic acid, quercetin, and rutin. All the tested polyphenols inhibited TPO; the IC_50_ values ranged from 0.004 mM to 1.44 mM (for rosmarinic acid and rutin, respectively). All these pure phytochemical substances exhibited different modes of TPO inhibition. Rutin and rosmarinic acid showed competitive, quercetin—uncompetitive and chlorogenic acid—noncompetitive inhibition effect on TPO. Homology modeling was used to gain insight into the 3D structure of TPO and molecular docking was applied to study the interactions of the inhibitors with their target at the molecular level. Moreover, the type and strength of mutual interactions between the inhibitors (expressed as the combination index, CI) were analyzed. Slight synergism, antagonism, and moderate antagonism were found in the case of the combined addition of the pure polyphenols. Rutin and quercetin as well as rutin and rosmarinic acid acted additively (CI = 0.096 and 1.06, respectively), while rutin and chlorogenic acid demonstrated slight synergism (CI = 0.88) and rosmarinic acid with quercetin and rosmarinic acid with chlorogenic acid showed moderate antagonism (CI = 1.45 and 1.25, respectively). The mixture of chlorogenic acid and quercetin demonstrated antagonism (CI = 1.79). All the polyphenols showed in vitro antiradical ability against 2,2′-azinobis-(3-ethylbenzothiazoline-6-sulfonic acid), ABTS. The highest ability (expressed as IC_50_) was exhibited by rosmarinic acid (0.12 mM) and the lowest value was ascribed to quercetin (0.45 mM).

## 1. Introduction

Thyroid peroxidase, also called thyroperoxidase (TPO, EC 1.11.1.1-14) or iodide peroxidase, is an enzyme participating in the synthesis of thyroid hormones. In the human body, it is encoded by the TPO gene located on chromosome *2p25* [1]. TPO catalyzes iodide oxidation to form iodine atoms, which are added onto tyrosine residues on thyroglobulin for the production of thyroxine or triiodothyronine, i.e., thyroid hormones [2]. The mechanism mentioned above can be described by reactions occurring in the following order: TPO is oxidized by H_2_O_2_ and then TPO can oxidize iodide ions. Oxidized iodide ions bind to tyrosyl residues of thyroglobulin (TG). Formation of T4 and T3 iodothyronines is an effect of oxidation and coupling of hormonogenic iodotyrosines [3].

As the TPO enzyme is a heme peroxidase, it cannot oxidize the substrate without having been oxidized. To oxidize TPO, the H_2_O_2_ molecule is necessary. The H_2_O_2_ molecule is generated only at the apical surface of thyrocytes, and TPO molecules present at this surface are activated [4].

**Figure 1:**
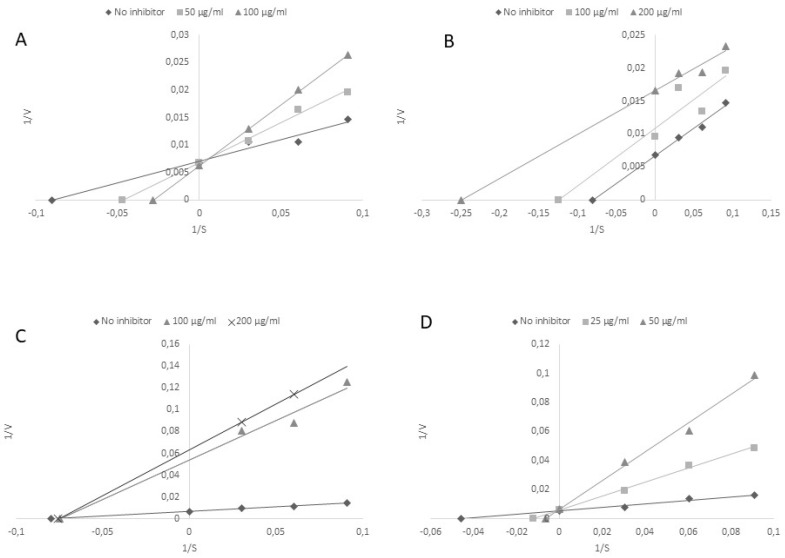
Lineweaver—Burk double reciprocal plots for the inhibition of TPO by rutin (**A**), quercetin (**B**), chlorogenic acid (**C**) and rosmarinic acid (**D**). Plots are expressed 1/velocity versus 1/guaiacol [µg/mL] without or with inhibitors in reaction solution.

TPO is organized as a 933-residues homodimer. The N-terminal propeptide (residues 1–108) is cleaved in the mature protein. Three domains in the TPO extracellular region (residues 109–846) display a high degree of sequence similarity to domains of known 3D structure: Myeloperoxidase (MPO)-like domain (residues 142–738), complement control protein (CCP)-like domain (residues 740–795), and epidermal growth factor (EGF)-like domain (residues 796–846), [5]. The transmembrane domain is constituted by residues 847–871 and intracellular domain by residues 872–933. Figure 1 presents the dimeric MPO-like domain in trans orientation with heme molecules exposed to thyroid follicular lumen. Le [5] considered also an alternative *cis* orientation, with heme molecules facing the thyrocyte membrane. According to the above studies cis orientation is slightly more energetically stable. However, other reported data suggested that in order to perform the oxidation and further iodination of thyroglobulin the catalytically active portion of the enzyme projects into the follicular lumen [6], which requires trans orientation of the dimeric MPO-like domain.

Thyroid peroxidase is the major antigen in human Hashimoto’s disease, and anti-TPO antibodies induce complement-dependent cytotoxicity. Furthermore, antibodies against complement (anti-C1q) are detected in patients with Hashimoto’s disease. They are correlated with thyroid-stimulating hormone (TSH) levels. Many patients with congenital hypothyroidism have problems related to synthesis or iodination of TG that are connected to TPO deficiency. When TPO activity is not normal or is totally absent, thyroid iodide organification may occur giving rise to congenital hypothyroidism. Another autoimmune disease correlated with pathological thyroid gland functioning is Grave’s disease. It is characterized by the presence of antibodies against the TSH receptor, thyroid peroxidase enzyme (TPO), and thyroglobulin (TG). TSH receptor antibodies play a crucial role in the development of hyperthyroidism. The increase in thyroid hormone synthesis is associated with the onset of Grave’s disease, showing T3 dominance [7]. Pathological conditions of the thyroid are associated with oxidative stress. Oxidative stress results mainly from excessive production of ROS, which are not removed by natural repair mechanisms. These mechanisms may be supported by substances with antioxidative activity, e.g., phytochemical compounds, especially phenolics, provided to the organism [8]. Thus, diet rich in food of plant origin is considered an important element of prevention of so called lifestyle diseases (for example Alzheimer’s disease, arteriosclerosis, cancer, cirrhosis, chronic obstructive pulmonary disease, diabetes, hypertension, heart disease, stroke). Many epidemiological studies have found that the consumption of foods and drinks with high phenolic content is associated with the prevention of coronary disease, cancer, etc. [9,10]. It is known that plants are a rich source of secondary metabolites (i.e., antioxidants) such as polyphenols with documented biological activity.

Combinations of different pure bioactive compounds or their extracts from food sources can increase the benefits of individual bioactive compounds. This type of interaction is referred to as synergism [11]. In some cases, mixtures of two compounds may lower the biological effects, e.g., they can decrease the capability to scavenge free radicals in comparison with this ability exhibited by single substances [12]. The available studies of the influence of food compounds on TPO activity are not sufficient and sometimes inconsistent, hence the need for additional, more complex investigation. The first step of the investigation should be to carry out an analysis in simple model systems; therefore, studies of pure chemical substances: Quercetin, chlorogenic acid, rutin and rosmarinic acid were carried out. Quercetin is a natural flavonoid present in many plants such as berries, onions, tea, or apples. It has anti-inflammatory, antioxidant, anti-apoptotic, and anticancer properties [13]. Studies reported by Cheng et al. [14] showed that quercetin could be used in the treatment of human retinal inflammatory diseases. Chlorogenic acid is present mainly in coffee and tea, as well as in grape wines, tropical fruits, cabbages, and root vegetables [15]. This polyphenolic acid plays a role in inducing apoptosis in chronic myeloid leukemia cells [16], glucose and lipid metabolism, inhibition of DNA methylation. It also has anti-obesity and anti-inflammatory activity, attenuates hypertension, and modifies the concentrations of cholesterol, triacylglycerols, and minerals [15]. Rutin is a non–toxic biologically active flavonoid. It is present in many plant products, such as tea, coffee, red wine, oil [17], and linden and heather honeys [15]. Rutin plays a role as an antioxidant mostly in free radical scavenging and in the treatment of hypertension, hemorrhoids, and spontaneous bleeding. It also protects vascular walls and shows antiviral and antimicrobial activity. Moreover, rutin has anti-inflammatory and anti-allergic properties [17]. Plants from the Lamiaceae family comprising many herbs: Rosemary, oregano, marjoram, sage, basil, thyme, and chia seeds are a source of rosmarinic acid [18]. Rosmarinic acid has a high antioxidant potential as well as anti-inflammatory, antiviral, antimicrobial, and antimutagenic activity [19].

In the natural environment, all substances appear not as single compounds but in connection with other substances. Due to this fact, our study includes the isobolographic analysis. This method is widely used for analysis of drug combinations and is also applied in the analysis of food compounds

The study is a part of more complex investigations, which focus on the influence of biologically active substances contained in food components on TPO activity. The obtained data can be useful in choosing an appropriate diet coordinated with the type of thyroid disorder (hyperthyroidism, hypothyroidism). The present study determines the effect of four most common dietary polyphenols (chlorogenic acid, rosmarinic acid, quercetin, and rutin) on TPO activity to elucidate these mechanisms.

## 2. Results and Discussion

### 2.1. Antioxidant Capacity

The antioxidant capacities of pure polyphenols (quercetin, rutin, rosmarinic acid, chlorogenic acid) were evaluated by the most commonly used antioxidant assay, i.e., ABTS method. The results clearly indicated that the polyphenols significantly scavenged free radicals in this assay. The ABTS radical scavenging ability of rosmarinic acid was found to be the highest. The lowest antiradical activity was observed for the quercetin (Table 1).

The results obtained by Grzesik et al. [20] showed antiradical properties in grapes (which are a source of quercetin) [21] for a solution concentration of 50 µg/mL at the level of 43.06% in the case of skin, 16.75%—seeds, and 7.01%—flesh. Chlorogenic acid exhibited ABTS radical scavenging activity at a IC_50_ value of 0.267 mM, which was higher than the results obtained for quercetin (0.447 mM) and rutin (0.292 mM) but lower than for rosmarinic acid (0.151 mM).

### 2.2. TPO Inhibitory Studies

The biochemical characteristic of TPO isolated from the thyroid gland is shown in Table 2.

Polyphenols showing inhibitory effect on TPO activity used in the study and IC_50_ value, which was calculated at fitted models as the concentration of the tested compound that gave 50% of the maximum response based on a dose-dependent mode of action were presented in Table 3.

All the tested polyphenols inhibited TPO, but with quite different potencies (Table 3). The EC50 values ranged from 0.004 mM to 1.44 mM with the following order of potency: Rosmarinic acid > rutin > quercetin > chlorogenic acid. Studies conducted by Divi and Doerge [23] showed a similar relationship between quercetin and rutin, where quercetin had a higher TPO inhibitory effect than rutin (IC_50_: 2.4 µM for quercetin and 40.6 µM for rutin).

Polyphenols are not the only substances, which can inhibit TPO activity. Amino acids are TPO inhibitors as well [24]. Scientists found that different amino acids inhibited TPO with different strength. Similar results were observed in the case of polyphenols.

It has been demonstrated that TPO inhibitors are potential therapeutic agents in hyperthyroidism. Pure substances were chosen on the basis of preliminary studies (unpublished data) and the main reason for choosing the polyphenols was their presence in plant food sources and the high antioxidant potential. The antioxidant potential is the main property of polyphenols, which show anticarcinogenic, antimutagenic, and antiaging activity, contained in medicinal plants [25]. The next step of our investigation was to determine the inhibitory potential of the tested polyphenols against TPO activity. As expected, the pure chemical solutions demonstrated high TPO inhibitory activity. The chosen polyphenols showed different modes of TPO inhibition. Rutin and rosmarinic acid acted as competitive inhibitors (Figure 1A,D). The uncompetitive mode of inhibition was determined for quercetin with respect to the guaiacol concentration, since Km and Vmax were affected (Figure 1B). The noncompetitive type of inhibition was indicated in the case of chlorogenic acid, where Vmax, but not Km, was affected (Figure 1C).

In our study, the IC_50_ value for quercetin was 0.199 mM, whereas data obtained by Divi and Doerge [23] showed that the IC_50_ value was 2.4 µM in relation to TPO. In our study, the IC_50_ value for rutin was 0.121 mM, whereas data obtained by Divi and Doerge [22] showed an IC_50_ value of 40.6 µM. The differences in these data can be associated with the differences in the methodology, solvents, and the source of TPO. Elucidation of the mechanisms observed in the analyzed polyphenols may provide important insight in the development and possibly prevention of inflammation.

Data obtained in this study indicate the ability of dietary polyphenols to inhibit TPO activity (Table 3). Of course, their ability to inhibit the activity of TPO is definitely lower than the commonly used drugs propylthiouracil (PPT) and methimazole (MMI) [22]. The major role in creating the biological activity of phenolic compounds plays their bioavailability, however it is still not fully understood. The intestinal absorption and metabolism of chlorogenic acids (385 μmol) following a single intake of 200 mL of instant coffee by human with an ileostomy was investigated by Stalmach et al. [26]. The HPLC–MS3 analysis of 0–24 h post-ingestion ileal effluent revealed the presence of 274 μmol of chlorogenic acids and their metabolites accounting for 71 of intake [26]. Quercetin is widely distributed in edible plants, mainly as glycosides such as rutin. It has been reported to be absorbed in mammals, but its metabolism needs further investigation to evaluate its possible physiological effects [27]. Hollman et al. [28] have found a human plasma concentration of about 0.65 umol/l quercetin after consumption of a meal containing 150 g of fried onions (equivalent to 64 mg of pure quercetin). Rutin was absorbed more slowly than quercetin because it must be hydrolysed by the cecal microflora. Following six weeks supplementation with rutin, significant changes in the plasma levels of quercetin, kaempferol and isorhamnetin were measured in the rutin-treated volunteers. The increase of 2.5 fold in plasma quercetin, 3-fold in plasma kaempferol and 10 fold in plasma isorhamnetin was found [29]. Rosmarinic acid (RA) exhibits diverse pharmacological effects, however, its oral absolute bioavailability and dose proportionality in vivo have not been comprehensively studied. The absolute bioavailability of RA in rats was estimated as 1.69%, 1.28% and 0.91% after oral administration of RA at the doses of 12.5, 25 and 50 mg/kg, respectively [30]. The serum concentration of total rosmarinic acid peaked at 1 h after administration of *Melissa officinalis* extract containing 500 mg rosmarinic acid in a fasted state, with a maximum serum concentration 162.20 nM [31]. In the light of the cited works, it seems difficult to achieve bioavailability at the IC_50_ level obtained in our study, therefore, the further part of the work is to determine of interaction between these compounds as a factor that can increase their effectiveness.

There are not many data about the influence of pure chemicals on TPO activity. The first data reports the influence of extracts from peanut seed coats [32]. The study showed the total polyphenol content in the plant material, but did not focus on the interactions between the substances. Attention was also paid to the inhibitory influence of the polyphenols contained in the peanut seed coat on the TPO activity. As shown by literature studies, peanuts are a rich source of dietary flavonoids [33]. In another study, the influence of the green tea extract on TPO activity was investigated. Green tea is a rich source of dietary polyphenols such as catechin, quercetin, kaemferol, and chlorogenic acid. It was found that green tea extracts inhibited TPO in a dose-dependent manner. Therefore, it was concluded that most of the polyphenols contained in tea extracts are potential inhibitors of TPO [32].

Many studies aimed to explore the influence of particular drug components on TPO activity and interactions between these components. Data concerning the influence of pure polyphenols on in vitro TPO activity are sparse. There are data on the influence of polyphenols on other enzymes e.g., xanthine oxidase, and lipoxygenase. Data obtained by Gawlik–Dziki et al. [34] showed the influence of pure chlorogenic and ferulic acid extracts on LOX activity. Lin [35] showed the ability of pure polyphenolic substances to inhibit xanthine oxidase and their mode of action. As described above, there are literature data confirming that polyphenolic compounds have an impact on enzymatic activity, but there are very limited data about the influence of pure polyphenolic substances on TPO activity.

To characterize further the binding region of TPO, the Lineweaver—Burk double reciprocal plots are shown in Figure 1; kinetic parameters were presented in Table 4. 

### 2.3. Molecular Modelling

In order to study the interactions of the considered inhibitors with TPO at the molecular level, the homology model of TPO was built. The homodimeric model with two molecules of heme bound in the MPO-domain is presented in Figure S1A.

**Figure 2:**
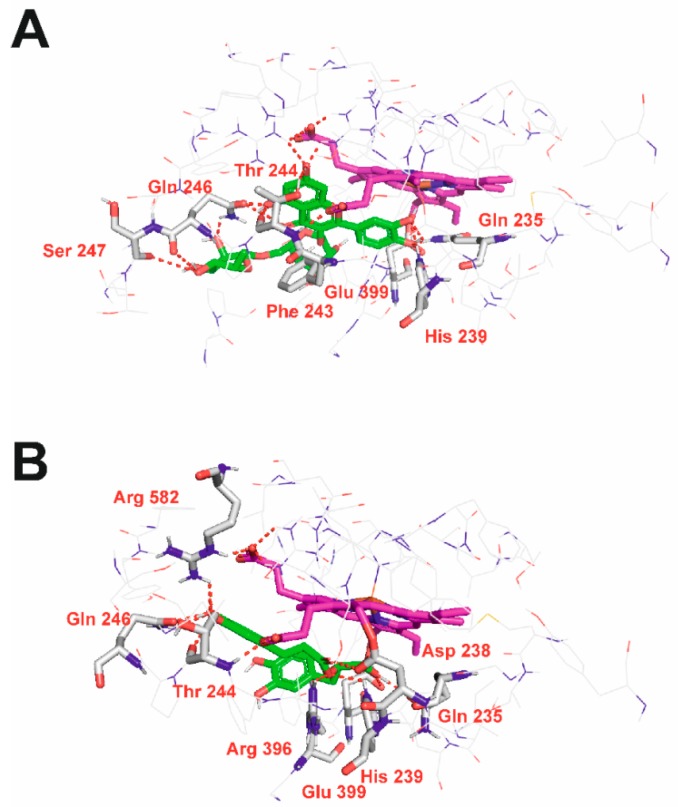
3D view of rutin (**A**) and rosmarinic acid (**B**) in the catalytic site of TPO. Inhibitor molecules shown as sticks with green carbon atoms. Heme shown as sticks with magenta carbon atoms. Protein presented in wire representation with grey carbon atoms. Most important residues shown as sticks. Hydrogen bonds depicted as red dashed lines. Non-polar hydrogen atoms omitted for clarity.

Competitive inhibitors, i.e., rutin and rosmarinic acid were docked to the catalytic site of TPO. In general, rutin was scored higher than rosmarinic acid in molecular docking simulations, which is in agreement with experimental data. The selected binding poses of these inhibitors are shown in Figure 2. Rutin (Figure 2A) forms hydrogen bonds with Gln 235, His 239, Phe 243, Thr 244, Gln 246, Ser 247 and Glu 399. Rosmarinic acid (Figure 2B) forms hydrogen bonds with Gln 235, Asp 238, His 239, Thr 244, Gln 246, Arg 396, Glu 399 and Arg 582. Comparison of Figure 2 and Figure S1 enables to conclude that both competitive ligands interact with residues involved in heme binding. Moreover, they block access of heme to His 239 in the catalytic site, making the catalytic process impossible.

**Figure 3:**
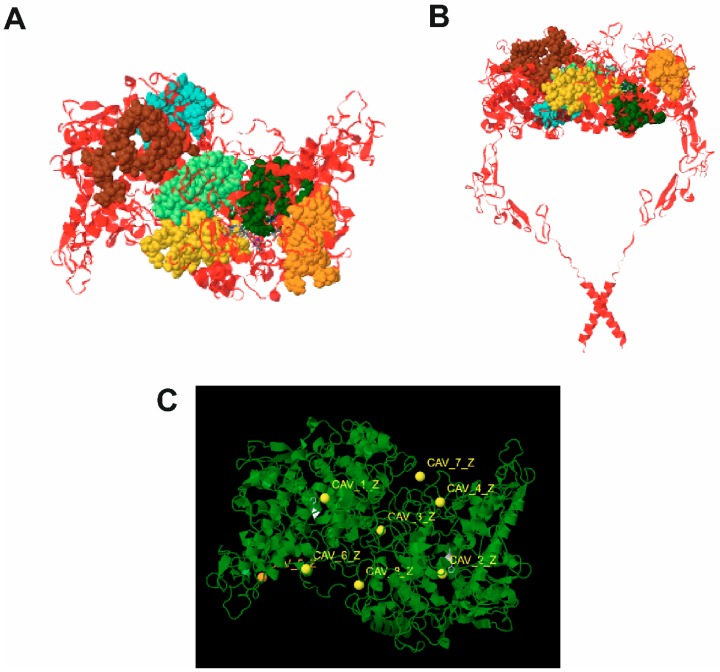
Prediction of allosteric sites at TPO using the normal mode analysis approach. (**A**) and (**B**) allosteric sites predicted using AlloPred on-line tool, top view and side view, respectively; (**C**) allosteric site predicted using PARS; all sites were found to affect protein flexibility but none of them was predicted as structurally conserved.

In order to find possible binding sites for binding of a non-competitive inhibitor chlorogenic acid and uncompetitive inhibitor quercetin AlloPred, PARS and Achilles on-line tools were used. Figure 3 presents possible allosteric binding sites of TPO predicted with AlloPred (Figure 3A,B) and PARS (Figure 3C) using the normal mode analysis approach. In particular both tools predicted that allosterism at TPO might be connected with ligand binding in the domain between protein subunits. Moreover, all allosteric sites predicted by PARS may affect protein flexibility and lead to a conformational change upon ligand binding but none of them was found to be structurally conserved.

Non-competitive inhibitor chlorogenic acid and uncompetitive inhibitor quercetin were docked using the blind docking approach so the whole protein was considered as a potential site for interaction. Uncompetitive inhibitors bind to the enzyme simultaneously as the substrate of the enzyme. The binding of the inhibitor influences the binding of the substrate, and vice-versa. The uncompetitive inhibitor usually binds to an allosteric binding site and exerts the allosteric effect on the active site by changing its conformation so that the affinity of the substrate for the active site is reduced. Non-competitive inhibitors bind to an allosteric site and change the conformation of the enzyme’s active site making it unsuitable for the substrate binding.

**Figure 4:**
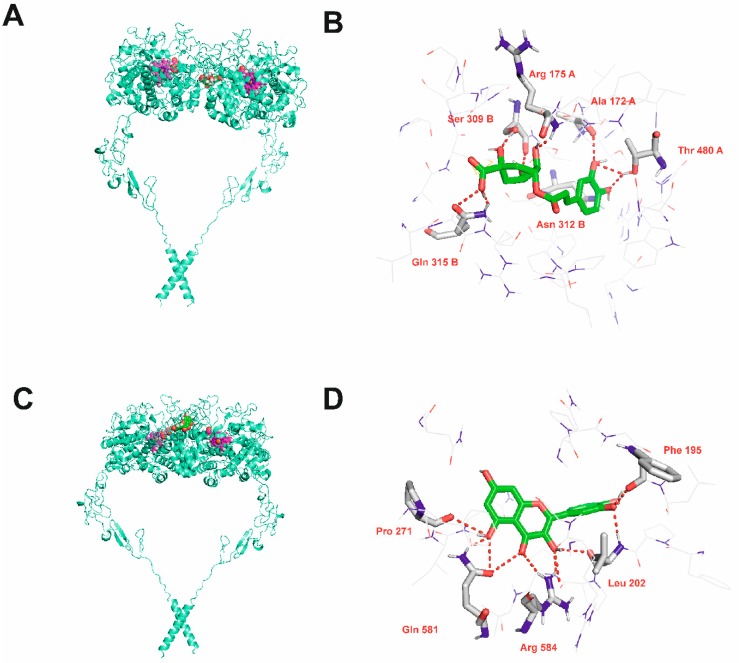
Results of blind docking of chlorogenic acid (**A**,**B**) and quercetin (**C**,**D**) to TPO. Parts A and C show general position of the binding site. Protein shown in cartoon representation. Heme and inhibitors shown as spheres with magenta and green carbon atoms, respectively. Parts B and D show details of the binding site. Inhibitor molecules shown as sticks with green carbon atoms. Protein presented in wire representation with grey carbon atoms. Most important residues shown as sticks. Hydrogen bonds depicted as red dashed lines. Non-polar hydrogen atoms omitted for clarity.

Figure 4 presents the results of blind docking of chlorogenic acid (Figure 4A,B) and quercetin (Figure 4C,D) to TPO. The identified binding pockets of both ligands were also found by AlloPred and PARS. Chlorogenic acid binding site was found in the binding pocket between the protein subunits. This inhibitor forms hydrogen bonds with Ala 172, Arg 175 and Thr 480 from one subunit and with Ser 309, Asn 312 and Gln 315 from the other subunit. Quercetin occupies a peripherial site within one subunit and forms hydrogen bonds with Phe 195, Leu 202, Pro 271, Gln 581 and Arg 584.

In order to find how binding chlorogenic acid in this pocket allosterically prevents TPO from heme binding, molecular dynamics simulations should be performed, which will be the subject of our future work.

### 2.4. Interactions Assay

It is known that polyphenolic substances contained in food sources appear in more complex combinations. Additionally, medicines with more than one active substance are more effective [4]. Therefore, the next step of the study was to estimate the type of interaction between the studied pure chemical standards (acting as TPO inhibitors). A method used for identification of the interactions between active compounds is the isobolographic analysis. This method is independent of the mechanism of activity; however, it should be emphasized that this analysis is quite complicated and labor consuming. The isobolographic analysis is a useful tool for determination of interactions between components of two-component mixtures, as well as those composed of plant extracts being mixtures of numerous active compounds (in the case of a linear relationship between the activity and sample concentration enabling determination of the IC_50_ value). The shape of the isobole gives information about the type of interaction and the CI value, i.e., the strength of interaction.

**Figure 5:**
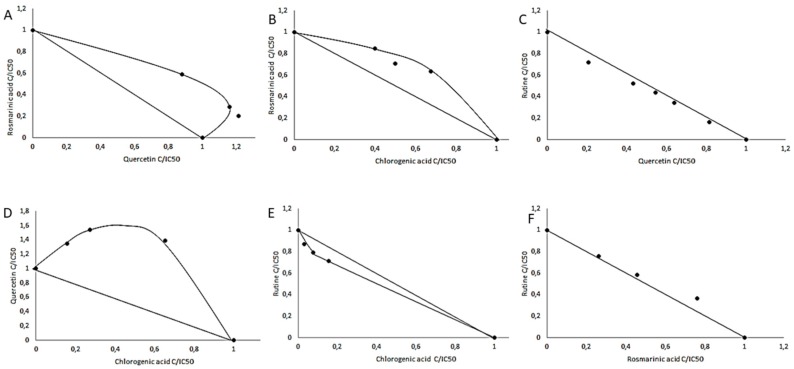
Dose-normalized isobolograms for chosen polyphenols with TPO inhibitory activity.

As presented in Figure 5, the interactions between the polyphenols used in the study were synergistic, antagonistic, and additive. Synergism means that two components mutually enhance their activities (concave isobole), antagonism means that two components decrease the activity of the single component (convex isobole), and additive interaction (the isobole is a straight line) [36].

The isobolographic analysis showed that the rutin and chlorogenic acid acted synergistically (Figure 5E), whereas additive interaction was found in the case of rutin and quercetin or rutin and rosmarinic acid (Figure 5C,F). Chlorogenic acid and rosmarinic acid showed antagonistic interaction (Figure 5B) as well as chlorogenic acid with quercetin and rosmarinic acid with quercetin (Figure 5D,A). All types of interactions were expressed as a CI (Combination index) value in accordance with the interpretation by Chou [36], which explains the strength of the interactions. As shown by the CI values, the pure chemicals showed moderate antagonism, antagonism, nearly additive action, and slight synergism (Table 5).

The isobolographic analysis is used for characterization of pharmaceuticals. There are no data on the influence of polyphenolic combinations on TPO activity. Some studies have shown the influence of phytochemical compounds on the activity of other enzymes, e.g., xanthine oxidase (XO) and lipoxygenase (LOX). Studies carried out by Gawlik-Dziki [37] demonstrated inhibitory activity of pure and dietary polyphenols on XO activity and interactions between these inhibitors. Other studies focused on the interactions of LOX and OX inhibitors derived from natural sources of dietary polyphenols [38].

## 3. Materials and Methods

### 3.1. Chemicals

Sucrose (α-d-glucopyranosyl-(1→4)-β-d-fructofuranoside), Tris (1,3-Propanediol-2-amino-2-hydroxymethyl), KCl, NaCl, MgCl_2_, 90% ethanol, NaOH, guaiacol (2-methoxyphenol), H_2_O_2_ (hydrogen peroxide), Bradford reagent, ABTS (2,2′-azinobis-(3-ethylbenzothiazoline-6-sulfonic acid), quercetin, rosmarinic acid, chlorogenic acid, rutin and Trolox (6-hydroxy-2,5,7,8-tetramethylchroman-2-carboxylic acid) were purchased from Sigma-Aldrich Company (Poznan, Poland). All other chemicals were of analytical grade.

### 3.2. Material

Porcine thyroid glands were obtained at a local slaughterhouse (Lublin, Poland) and stored in −20 °C until used.

### 3.3. Preparation of Pure Substance Solutions

Chlorogenic acid, quercetin, rosmarinic acid, and rutin were diluted to concentrations of 25 µg/mL (only for rosmarinic acid), 50 µg/mL, 100 µg/mL, 200 µg/mL and used for further assay.

### 3.4. In Vitro Antioxidant Capacity Assay

The ABTS radical scavenging activity was determined according to Re et al. [39] with some modifications. 250 µL of ABTS solution was mixed with 10 µL of each pure polyphenols solutions (concentration 50 µg/mL) and measured at the wavelength 724 nm using a UV/Vis spectrophotometer (BioTek, Model Epoch2TC, Winooski, Vermont, VT, USA) after 15 min of incubation in room temperature. The inhibition percentage of ABTS discoloration was calculated using the following equation:

$$\mathrm{AA}=\frac{A_{c}-A_{p}}{A_{c}}\times 100\%$$

Where A_c_—the absorbance of control, A_p_—the absorbance of pure polyphenols solutions.

### 3.5. TPO Preparation

The assay was prepared according to Jomaa [22] with some modifications. The frozen thyroid gland was cut into slices and homogenized in a buffer containing 0.25 M sucrose, 2 mM Tris—HCl, 100 mM KCl, 40 mM NaCl, 10 mM MgCl_2_ (pH 7.4) with a Philips homogenizer. The thyroid gland was than centrifuged two times at 4000 RPM per 15 min in temperature +4 °C. The enzyme protein was then salted-out to 60%. The supernatant was used for further analysis and was stored in a −20 °C freezer.

Protein content was determined according to the Bradford [40] with some modification. The measurement was made using a plate spectrophotometer (BioTek, Model Epoch2TC, Winooski, Vermont, VT, USA) in 96–well plates at a wavelength of 595 nm. In the single well 10 µL of the sample probe and 250 µl of Bradford reagent were mixed. The measurement was made with three repetitions.

The activity of the enzyme was assayed using a guaiacol assay. The reaction mixture contained: 33 mM guaiacol, 0.27 mM H_2_O_2_, and 33 mM of prepared sucrose buffer. The reaction components were incubated in 37 °C before the assignment. The absorbance was determined using a Shimadzu spectrophotometer (Model UV-1280, Shimadzu Corporation, Kyoto, Japan) at a wavelength of 470 nm. The assay was conducted as follows: To a cuvette, 180 µL of buffer, 100 µL of guaiacol and 40 µL of TPO were mixed to the final volume of 420 µL. The cuvette was then placed into the spectrophotometer and the reaction was started by the addition of 100 µL H_2_O_2_. Absorbance readings were recorded every minute for a total of 3 min. Verification of the TPO activity was accomplished by linearly correlating the TPO concentration with absorbance readings.

### 3.6. TPO Inhibitory Assay

The assay was used according to Jomaa [22] with some modification. The measurement was made using a plate spectrophotometer (BioTek, Model Epoch2TC, Winooski, Vermont, VT, USA) in 96–well plates at a wavelength of 470 nm. The assay was conducted as follows: 50 µl of buffer, 40 µl of pure substance solution, 50 µL of guaiacol, 20 µL of TPO enzyme and 50 µL H_2_O_2_. The total volume of the mixture was 210 µL. In the sample probe, extracts were replaced by the buffer. Absorbance readings were recorded every minute for a total of 3 min in 37^o^. As a unit of TPO activity is defined as the change of absorbation per minute.

TPO inhibitory activity was calculated as follows:

$$\%inhibition=\left(1-\frac{\frac{\Delta A}{\min_{\mathrm{test}}}}{\Delta {\mathrm{Amin}}_{\mathrm{blank}}}\right)\times 100$$

Where: ΔA/min test is the linear absorbance change per minute of the test material and ΔA min blank is the linear change in absorbance per minute of the blank.

The IC_50_ value was determined by the interpolation of dose response curves. The IC_50_ values were calculated at fitted models as the concentration of the tested compound that gave 50% of the maximum inhibition based on a dose-dependent mode of action. The mode of inhibition of the enzyme was performed using the Lineweaver–Burk plot.

### 3.7. Molecular Modelling

The protein sequence of human TPO was extracted in a FASTA format from the UniProt database (Entry ID: P07202) [41]. Searching for templates was performed using the NCBI BLAST web server (https://blast.ncbi.nlm.nih.gov/). The MPO-like, CCP-like and EGF-like domains as well as a transmembrane domain were modelled with the homology modelling approach using five different templates. The MPO-like domain was modelled applying X-ray crystal structures of human myeloperoxidase at a 1.8 A resolution (PDB ID: 1CXP as a template [42] (residues 142–738 of TPO modelled using residues 167–744 of the template, sequence identity: 47%). The CCP-like domain was modelled applying X-ray structures of N-terminal domain of complement factor H-related protein at a 1.99 Å resolution (PDB ID: 3ZD2) as a template [43] (residues 730–795 of TPO were modelled using residues 57–123 of a template, sequence identity: 38%). The EGF-like domain was modelled applying X-ray structures of EGF-like module containing mucin-like hormone receptor-like 2 precursor at a 2.6 Å resolution (PDB ID: 2BO2) [44] and venom prothrombin activator pseutarin-C non-catalytic subunit at a 3.32 Å resolution (PDB ID: 4BXS) [45] as templates (residues 794–830 of TPO were modelled using residues 41–77 of 2BO2, sequence identity: 41%; residues 814–844 of TPO were modelled using residues 100–130 of 4BXS, sequence identity: 42%). The transmembrane domain was modelled applying a solution NMR structure of receptor tyrosine-protein kinase erbB-4 dimeric membrane domain (PDB ID: 2L2T) as a template [46] (residues 851–871 of TPO were modelled using residues 647–667 of a template as previously reported [5]. A population of 150 homology models of TPO was generated using Modeller 9.19 [47]. The symmetry of the homodimer was maintained by appropriate constraints in the Modeller software. Constraints were also used to model the inter-subunit disulfide bridge (at Cys 296). Residues 841–847 were modelled applying the appropriate loop modelling protocol of the Modeller software. Secondary structure constraints were applied to assure the α-helical structure of the protein transmembrane part. Heme molecule was modelled using Modeller.

The final models were evaluated based on their Discrete Optimized Protein Energy (DOPE) profiles obtained from the Modeller software. Twenty models with lowest DOPE values were further validated using Verify3D [48], ANOLEA (Atomic Non-Local Environment Assessment) [49] and ProCheck [50]. The best model was properly protonated with H++ server [51]. Next, the final TPO model was minimized using 500 steps minimization with Gromacs v. 5.0.7 [52] applying the Amber03 force field.

The 3D structures of quercetin, rutin, rosmarinic acid and chlorogenic acid were modelled with the Hartree-Fock approach and 6-31G* basis set of Spartan v. 10 VI.0.1 (Spartan 10 VI.01 (2016) Wavefunction, Inc., Irvine). Molecular docking of competitive ligands was carried out using the Molegro Virtual Docker 6.0 software for docking simulations of flexible ligands into the rigid TPO model. The docking space was limited and centered around the heme molecule for competitive inhibitors. The actual docking simulations were performed using the following settings: Number of runs = 100; maximal number of iterations = 10,000; maximal number of poses = 50, and the poses representing the lowest value of the scoring function (MolDockScore) were further analyzed as previously reported [53]. In order to identify the potential binding sites for a non-competitive ligand and an uncompetitive ligand, AlloPred [54] and PARS [41] on-line tools were used. AlloPred uses a novel method of normal mode analysis and pocket features to predict allosteric pockets on proteins. PARS is also based on the normal mode analysis and constitutes a simple and fast method that queries protein dynamics and structural conservation to identify pockets on a protein structure that may exert a regulatory effect upon the binding of a small-molecule ligand. The achilles blind docking on-line tool [54] was used to find possible binding poses of a non-competitive and an uncompetitive ligand.

Visualization of molecular modelling results was achieved with the Maestro Release 2019.1 (Small-Molecule Drug Discovery Suite 2019-1, Schrödinger, LLC, New York, NY, USA, 2019) and PyMol 2.0.4 (The PyMOL Molecular Graphics System, Version 2.0 Schrödinger, LLC, New York, NY, USA) software.

### 3.8. Effect of Pure Substances Combinations on TPO Activity (Isobolographic Analysis)

Results (type and strength of interactions) can be shown on the isobolograms and described by the combination Index (CI). When CI is lower than one, indicates synergy; when CI is equal to one, indicates addition; when CI is higher than one, indicates antagonism [36]. Isobolograms were performed according to Chou [36]. For this assay only substances with 100 µg/mL concentrations were used (according to previous results). Pure substances were mixed in various volume ratios: 1:4, 4:1, 3:2, 2:3, 1:1. All of the mixtures were made in combinations of two substances. The inhibitory assay was made using the same proportions as with single solutions. The evaluation of the interaction was done using the combination index (CI) equation for n-drug combination at an x% inhibition as follows:

$$\mathrm{CI}=\frac{{\left(D\right)}_{1}}{{\left(D_{x}\right)}_{1}}+\frac{{\left(D\right)}_{2}}{{\left(D_{x}\right)}_{2}}=\frac{1}{{\left(\mathrm{DRI}\right)}_{1}}+\frac{1}{{\left(\mathrm{DRI}\right)}_{2}}$$

Where CI—sum of the dose of drugs that exerts x% when combined; D_x_—for D as a single substance that inhibits a system ×%. CI value shows the type of interactions, which can be synergistic, antagonistic or additive, when its value is smaller, greater or equal to one.

### 3.9. Statistics

Experimental data were presented as a means ± S.D. For biochemical analyses and means ± SEM for anticancer activity assays. In biochemical analyses, statistical significance was estimated with Tukey’s test (for the data obtained from three independent samples of each extract in three parallel experiments; *n* = 9).

## 4. Conclusions

Our investigation was mainly focused on the interactions between pure polyphenolic inhibitors of TPO in simple models where single polyphenols were applied.

In this study, simple combinations of substances were used to visualize the type of interactions between the food components and their inhibitory influence on the TPO enzyme and thus on changes in biological properties. The most effective TPO inhibitor as well as the compound capable of scavenging free radicals is the rosmarinic acid.

We performed molecular modeling studies of ligand-inhibitor interactions to illustrate and clarify their competitive, uncompetitive, or non-competitive modes of action at the molecular level. In the study, we found nearly additive interactions between rutin and quercetin and between rosmarinic acid and rutin. Slight synergism was observed between the chlorogenic acid and rutin, whereas moderate antagonism was detected between rosmarinic acid and quercetin and between rosmarinic acid and chlorogenic acid. There was antagonism between chlorogenic acid and quercetin. More research should be undertaken to understand completely the mechanism of interactions between bioactive compounds and their influence on TPO activity. It seems difficult to achieve bioavailability at the IC_50_ level obtained in our work (Table 3), more importantly is the knowledge about the interactions of these compounds. As has been shown, they substantially affect their potential inhibitory activity (Figure 5, therefore the effect of the mixture of inhibitors can be substantially different from the predicted and even lower concentrations can be effective. An explanation of these relationships is extremely difficult and requires further, extensive and interdisciplinary research.

## Figures and Tables

**Table 1:** Chemical structure and ABTS (2,2′-azinobis-(3-ethylbenzothiazoline-6-sulfonic acid) radical scavenging properties of chosen polyphenols (quercetin, rutin, chlorogenic acid, rosmarinic acid) expressed in IC_50_ (Efficient Concentration), *n* = 9.

Compound	Chemical Formula	IC_50_ [mM]
quercetin	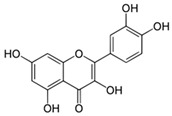	0.447 ± 0.018 e *
rutin	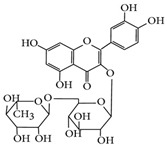	0.292 ± 0.010 c
chlorogenic acid	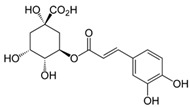	0.267 ± 0.008 b
rosmarinic acid	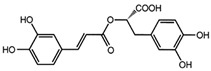	0.151 ± 0.006 a
Trolox (positive control)	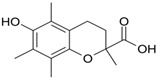	0.407 ± 0.014 d

* The values designated by the different letters are statistically significantly different.

**Table 2:** Km value, Vmax and content of proteins in thyroid peroxidase (TPO) enzyme solution prepared from porcine thyroid gland, (*n* = 9).

Parameter	Value
Proteins [mg/mL]	0.67 ± 0.02
Km [μg/mL]	11.07 ± 0.47
Vmax [ΔAU/min]	140.85 ± 5.22

**Table 3:** IC_50_ [µM] value of polyphenols showing inhibitory effect on TPO activity used in the study, *n* = 9.

Compound	IC_50_ [mM]
Rutin	0.122 ± 0.004 c **
Rosmarinic acid	0.004 ± 0.00014 a
Quercetin	0.199 ± 0.008 d
Chlorogenic acid	1.439 ± 0.041 e
Propylthiouracil (PTU, positive control) *	0.004 a
Methimazole (MMI, positive control) *	0.0107 b

* According to literature data [22]; ** The values designated by the different letters are statistically significantly different.

**Table 4:** Mode of inhibition, K_i_ and V_max_ values of chosen polyphenols on TPO activity, *n* = 9.

Compound	Mode of Inhibition	Ki [mM]	V_max_ [ΔAU/min]
Chlorogenic acid	noncompetitive	0.17 ± 0.005 d *	149.25 ± 5.97 a *
Quercetin	uncompetitive	0.02 ± 0.001 b	149.25 ± 4.35 a
Rosmarinic acid	competitive	0.001 ± 0.00 a	175.44 ± 7.01 b
Rutin	competitive	0.13 ± 0.003 c	149.25 ± 5.01 a

* The values designated by the different letters are statistically significantly different.

**Table 5:** CI (Combination Index) value between polyphenolic mixtures. Scale according to Chou, 2006 [39], *n* = 9.

Compound	Quercetin	Rutin	Chlorogenic Acid	Rosmarinic Acid
Quercetin	-	0.096 ± 0.05 Nearly additive	1.79 ± 0.2 Antagonism	1.45 ± 0.03 Moderate antagonism
Rutin	0.096 ± 0.05 Nearly additive	-	0.88 ± 0.02 Slight synergism	1.06 ± 0.05 Nearly additive
Chlorogenic acid	1.79 ± 0.2 Antagonism	0.88±0.02 Slight synergism	-	1.25 ± 0.05 Moderate antagonism
Rosmarinic acid	1.45 ± 0.03 Moderate antagonism	1.06 ± 0.05 Nearly additive	1.25 ± 0.05 Moderate antagonism	-

## Supplementary Materials

The following are available online, Figure S1: A—Homology model of TPO. Protein shown in cartoon representation with subunits colored light and dark blue. Disulfide bridges depicted as spheres with yellow sulfur atoms. Heme molecules in the MPO domain presented as spheres with magenta carbon atoms. B—The Ramachandran plot for the homology model of TPO. Glycine residues shown as triangles and proline residues as squares. C—3D view of heme binding site at TPO. Heme molecule shown as sticks with magenta carbon atoms. Protein presented in wire representation with grey carbon atoms. Most important residues shown as sticks. Hydrogen bonds depicted as red dashed lines. Non-polar hydrogen atoms omitted for clarity.
