# Supplementary material for: Thyroid Peroxidase Activity is Inhibited by Phenolic Compounds—Impact of Interaction
Source: Molecules. 2019 Jul 30;24(15):2766. doi: 10.3390/molecules24152766 (PMC6696198; doi:10.3390/molecules24152766)
Supplement: Supplementary file 1 [file molecules-24-02766-s001.pdf]

Supplementary Information

Thyroid peroxidase activity is inhibited by phenolic compounds – impact of interaction

Ewa Habza-Kowalska[1], Agnieszka A. Kaczor [2,3], Justyna Żuk[2], Dariusz Matosiuk[2], Urszula Gawlik-Dziki[1*]

[1] Department of Biochemistry and Food Chemistry, University of Life Sciences, Skromna Str. 8, 20-704 Lublin, Poland, ewa.habza@student.up.edu.pl

[2] Department of Synthesis and Chemical Technology of Pharmaceutical Substances, Faculty of Pharmacy with Division of Medical Analytics, Medical University of Lublin, 4A Chodzki St., PL-20093 Lublin, Poland,

[3] School of Pharmacy, University of Eastern Finland, Yliopistonranta 1, P.O. Box 1627, FI-70211 Kuopio, Finland

Details of TPO homology model

Each subunit of TPO contains eleven disulfide bridges: Cys 142-Cys 158, Cys 259-Cys 269, Cys 263-Cys 286, Cys 375-Cys 389, Cys 598-Cys 655 and Cys 696-Cys721 in the MPO-like domain, Cys 742-Cys 756 and Cys 768-Cys 794 in the CCP-like domain while Cys 800-Cys 814, Cys 808-Cys 823 and Cys 825-Cys 838 in the EGF-like domain (Fig. S1 A). In comparison to the predicted data in the UniProt database, three additional intrasubunit disulfide bridges were found in the model: one in the MPO-like domain and two in the CCP-like domain. Two subunits are joined by the additional intersubunit disulfide bridge at Cys 296 (Cys 153 in the MPO template) as described previously [1]. The quality of the TPO homology model was evaluated using a few tools (see Materials and Methods for details). Fig. S1 B presents the Ramachandran plot for the TPO model which can be used to assess the stereochemical quality of the protein structure. The Ramachandran plot visualizes dihedral angles ψ against ϕ of amino acid residues in protein structure as many combinations of angles in a polypeptide chain are forbidden because of steric collisions between atoms. The Ramachandran plot confirms a good quality of the model as most residues in the forbidden regions are glycines or prolines.

TPO belongs to peroxidases which are heme-containing enzymes that use hydrogen peroxide as the electron acceptor to catalyze a number of oxidative reactions. The heme molecule is bound in a heme cavity which is deeply buried inside the structure of TPO (Fig. S1 A), similarly as reported for other members of heme peroxidase family [2]. Fig. S1 C shows details of the heme binding at the TPO. The model is in accordance with the current understanding of heme peroxidase functioning as there are two covalent ester linkages between heme group and protein involving conserved glutamate (Glu 399) and aspartate (Asp 238) residues with 1-methyl and 5-methyl groups of pyrrole rings A and C respectively of the heme moiety [3]. The heme iron of TPO has five coordination bonds: four equatorial with nitrogen atoms of tetrapyrrole ring, and the fifth heme ligand on the proximal heme side (axial bond) is a highly conserved imidazole ring of histidine residue (His 494) linking the heme to the protein. On the distal heme side (free axial coordination bond), heme can bind a water molecule. This H_2O molecule is replaced by H_2O_2 upon activation of a peroxidase [4]. Another histidine residue on the distal heme site (His 239) takes places in the catalysis process, most probably as a proton donor. In addition, side chains of Arg 491 and Arg 582 as well as the main chain of Thr 244 form hydrogen bonds wth carboxylic groups of heme (Fig. S1 C).

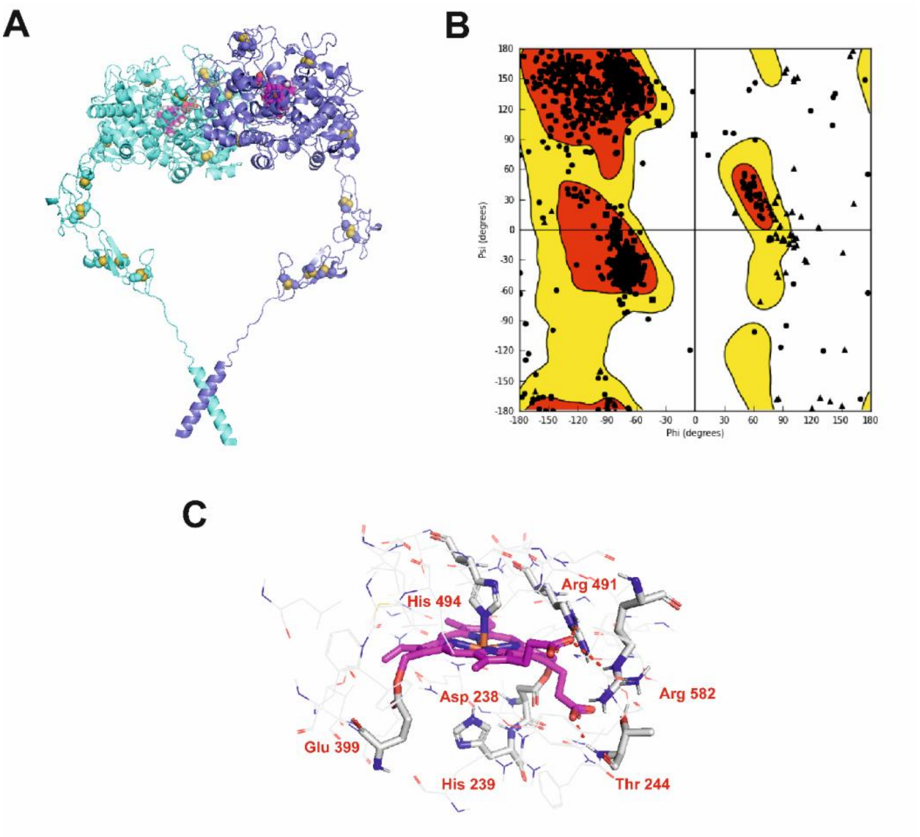

Fig. S1. A – Homology model of TPO. Protein shown in cartoon representation with subunits colored light and dark blue. Disulfide bridges depicted as spheres with yellow sulfur atoms. Heme molecules in the MPO domain presented as spheres with magenta carbon atoms. B – The Ramachandran plot for the homology model of TPO. Glycine residues shown as triangles and proline residues as squares. C – 3D view of heme binding site at TPO. Heme molecule shown as sticks with magenta carbon atoms. Protein presented in wire representation with grey carbon atoms. Most important residues shown as sticks. Hydrogen bonds depicted as red dashed lines. Non-polar hydrogen atoms omitted for clarity.

References

1. Le, S.N.; Porebski, B.T.; McCoey, J.; Fodor, J.; Riley, B.; Godlewska, M.; Góra, M.; Czarnocka, B.; Paul Banga, J.; Hoke, D.E.; et al. Modelling of thyroid peroxidase reveals insights into its enzyme function and autoantigenicity. *PLoS One* **2015**, *10*.

2. Sharma, S.; Singh, A.K.; Kaushik, S.; Sinha, M.; Singh, R.P.; Sharma, P.; Sirsohi, H.; Kaur, P.; Singh, T.P. Lactoperoxidase: Structural insights into the function, ligand binding and inhibition. *Int. J. Biochem. Mol. Biol.* 2013, *4*, 108–128.

3. Singh, P.K.; Iqbal, N.; Sirohi, H. V.; Bairagya, H.R.; Kaur, P.; Sharma, S.; Singh, T.P. Structural basis of activation of mammalian heme peroxidases. *Prog. Biophys. Mol. Biol.* 2018, *133*, 49–55.

4. Vlasova, I.I. Peroxidase Activity of Human Hemoproteins: Keeping the Fire under Control. *Molecules* **2018**, *23*.
